# Enhanced neurologic concept recognition using a named entity recognition model based on transformers

**DOI:** 10.3389/fdgth.2022.1065581

**Published:** 2022-12-08

**Authors:** Sima Azizi, Daniel B. Hier, Donald C. Wunsch II

**Affiliations:** ^^1^^ Applied Computational Intelligence Laboratory, Department of Electrical & Computer Engineering, Missouri University of Science & Technology, Rolla, MO, United States; ^^2^^ Department of Neurology and Rehabilitation, University of Illinois at Chicago, Chicago, IL, United States; ^^3^^ National Science Foundation, ECCS Division, Arlington, VA, United States

**Keywords:** named entity recognition, clinical concepts, concept extraction, phenotype, transformers, natural language processing, annotation

## Abstract

Although deep learning has been applied to the recognition of diseases and drugs in electronic health records and the biomedical literature, relatively little study has been devoted to the utility of deep learning for the recognition of signs and symptoms. The recognition of signs and symptoms is critical to the success of deep phenotyping and precision medicine. We have developed a named entity recognition model that uses deep learning to identify text spans containing neurological signs and symptoms and then maps these text spans to the clinical concepts of a neuro-ontology. We compared a model based on convolutional neural networks to one based on bidirectional encoder representation from transformers. Models were evaluated for accuracy of text span identification on three text corpora: physician notes from an electronic health record, case histories from neurologic textbooks, and clinical synopses from an online database of genetic diseases. Both models performed best on the professionally-written clinical synopses and worst on the physician-written clinical notes. Both models performed better when signs and symptoms were represented as shorter text spans. Consistent with prior studies that examined the recognition of diseases and drugs, the model based on bidirectional encoder representations from transformers outperformed the model based on convolutional neural networks for recognizing signs and symptoms. Recall for signs and symptoms ranged from 59.5% to 82.0% and precision ranged from 61.7% to 80.4%. With further advances in NLP, fully automated recognition of signs and symptoms in electronic health records and the medical literature should be feasible.

## I. Introduction

Several factors have accelerated interest in the automated recognition of clinical concepts in unstructured text held in electronic health records and electronic publications (1). First, most paper medical records have been converted to electronic health records (EHRs) (2) with as much as 80% of the data held as unstructured text (3). Second, most medical journals are available electronically (4). Third, the deep phenotyping and precision medicine initiatives have made the detailed description of patient signs and symptoms a key piece of data (5,6). Fourth, automated clinical concept recognition is an important area of natural language processing (NLP) research. Automated concept recognition is closely related to the NLP problems of text mining and named entity recognition. Other important NLP research areas include machine translation, text classification, text clustering, speech recognition, question answering, text summarization, sentiment analysis, picture captioning, and natural language understanding (7–14).

Krauthammer and Nenadic (1) have divided concept recognition (variously called term identification, concept extraction, and information extraction) into three steps: term recognition (identification of the text span corresponding to the clinical concept), term classification (identification of the class membership of the term, i.e., drug, disease, sign, symptom, etc.), and term mapping (linking of the term to an entry in a standard vocabulary with an identification code which is also known as “concept normalization” (15)). Clinical concept recognition is closely related to the NLP problem of named entity recognition (NER) in which text spans referring to named entities (people, places, organizations, etc.) are tagged and mapped to dictionaries, gazetteers, or other registries (16).

Text spans that encode clinical concepts (diseases, drugs, signs, symptoms, etc.) can be mapped (normalized) to hierarchical ontologies that include SNOMED CT with 352,000 concepts, the Human Phenotype Ontology (HPO) with 20,000 concepts, the Online Mendelian Inheritance in Man ontology (OMIM) with 97,000 concepts, or the UMLS Metathesaurus with 4.6 million concepts (17–20). The NLM UMLS Metathesaurus maintains interchangeable machine-readable codes for SNOMED CT, UMLS, HPO, and the OMIM.

Initial NER systems for clinical concept recognition were either dictionary-based, or rule-based (1,21,22). Some second-generation NER systems were based on machine learning algorithms such as conditional random fields, support vector machines, and hidden Markov models (23,24). Other second-generation NER systems developed as an outgrowth of advances in semantic and syntactic analysis (25,26). MetaMap utilizes linguistic analysis and statistical algorithms to identify clinical concepts in unstructured text and maps them to machine-readable codes in the UMLS (27,28). The UMLS has grown from 900,000 concepts, and 2 million names in 2004 (29) to 4.6 million concepts and 17 million names in 2022 (20). MetaMap tokenizes text input, finds sentence boundaries, and uses lexical and syntactic analysis to identify candidate phrases for mapping to concepts in the UMLS. Candidate phrases are compared to target strings in the UMLS, lists of potential clinical concepts are generated, and scored by statistical algorithms. MetaMap can recognize abbreviations, acronyms, and negation, can generate word variants, and can perform word sense disambiguation (27). In a preliminary study, we found that MetaMap can identify signs and symptoms in neurological case histories with an accuracy of 55–84% (30). Most MetaMap errors were false negatives due to a failure to recognize neurological concepts that had been expressed as descriptions (e.g., *reflexes were absent*) as opposed to those expressed as discrete lexical items (e.g., *hyporeflexia*). In their 2017 literature review of automated information extraction, Wang et al. (31) reviewed 263 information extraction studies and found most centered on identifying diseases or drugs. The most common systems used were MetaMap, MedLEE, and cTAKES (32–36) followed by traditional machine learning algorithms (conditional random fields, support vector machines, random forests, decision trees, and naive Bayes).

Third-generation systems for NER are built on deep learning (37–40). Lample et al. suggested a model for named entity recognition based on an RNN (recurrent neural network) with bidirectional LSTM (long short term memory) and conditional random fields (CRFs). Vani et al. (41) proposed a “grounded” RNN to predict medical diagnoses based on text from patient discharge summaries. Liu et al. (42) found that on a task to label protected health information in medical records that RNNs based on bidirectional LSTM outperformed those that used CRFs. An LSTM NER model with conditional random fields (CRFs) has been used to identify five classes of chemicals, species, genes/proteins, cell lines, and diseases (43). Hybrid methods that combine rule-based and machine learning-based methods have been proposed to identify protected health information (PHI) in clinical discharge summaries (44). Liu et al. (42) developed a hybrid system to identify clinical information by ensemble learning that combined the instances predicted from a bidirectional LSTM, a CRF model, and a rule-based system (45,46). Gehrmann et al. (47) used a convolutional neural network (CNN) for ten phenotyping tasks and compared it with other common NLP models. Arbabi et al. (48) have created a neural concept recognizer (NCR) that uses CNNs and word embedding to recognize clinical concepts in unstructured text. The NCR uses an encoder to convert input phrases to word vectors and word embedding to convert entries in the target ontology into word vectors. The similarity between the input phrases and concepts in the target ontology is calculated by the dot product. For concept recognition in PubMed abstracts or clinical notes, the NCR outperformed the NCBO Annotator and BioLark (49). RNNs and variants can handle long-term dependency in text, but only for a limited span length. The deep learning architecture transformers can process longer text spans and has shown improved performance on NLP tasks (50). Bidirectional encoder representations from transformers (BERT) have outperformed other neural network architectures on named entity recognition (51,50). For clinical concept recognition, BERT models that are pre-trained on the medical literature (BioBERT) or clinical notes (ClinicalBERT) outperform BERT models pre-trained on general corpora by at least 1% (52–55).

### A. Proposed approach

Although considerable work has been done on automated concept identification of drugs and diseases, less work has been done on the automated identification of signs and symptoms (52). Identifying signs and symptoms is critical to precision medicine and deep phenotyping (56). To make the problem tractable, we limited the signs and symptoms to the specialty of neurology and restricted the target ontology to a neuro-ontology with 1,600 concepts (57). Automating the recognition of signs and symptoms is more challenging than automating the recognition of diseases or drugs for three reasons. First, many neurological signs and symptoms have multiple synonyms; something that is not typical with diseases or drugs. For example, an expressionless face may be described as a “masked face,” or “hypomimia.” Second, physicians variably choose to record signs and symptoms as *descriptions* or as *names*. For example, a patient with diplopia can be described as “seeing double” or a patient with nausea can be described as “sick to their stomach.” In contrast, physicians uniformly identify drugs and diseases by name and not by description. Third, the meaning of a term may depend on context. For example, to a neurologist *ptosis* is a droopy eyelid, but to a gynecologist, *ptosis* is a prolapsed uterus.

**Figure 1:**
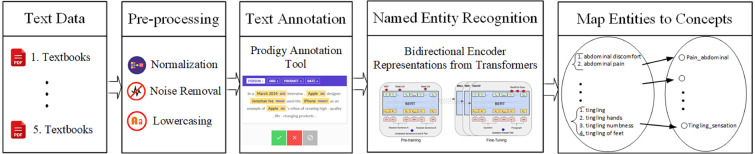
Overview of the pipeline that recognizes text spans that are clinical concepts in three corpora: Textbook neurology case histories, EHR physician notes, and Clinical synopses in the OMIM. Text spans are normalized by mapping to clinical concepts in a neuro-ontology.

We propose to identify and normalize the neurological signs and symptoms found in the unstructured text in two steps: first, we have trained a neural network-based named entity recognition model to identify text spans that contain clinical concepts (signs and symptoms). Second, we have normalized identified text spans by mapping them to clinical concepts in a neuro-ontology using a look-up table and similarity metric (Figure 1).

Since neurologic signs and symptoms can be extracted from both the medical literature and electronic health records, we have tested the concept identification pipeline on three corpora: case histories from neurological textbooks, neurological clinical synopses from the Online Mendelian Inheritance of Man (OMIM), and physician neurological notes from an electronic health record. With this work, we propose to address four questions:

1. Does writing style differ by corpus?
2. Does the accuracy of concept recognition differ by corpus?
3. Is the accuracy of clinical concept recognition reduced with longer text spans?
4. Does concept recognition based on BERT outperforms concept recognition based on CNNs?

Although the superiority of BERT over other neural networks for concept identification is well-established, the contribution of this work is to demonstrate that the accuracy of concept identification depends upon text span length and corpus writing style.

## II. Methods

### A. Corpora

We identified signs and symptoms (clinical concept identification) in three corpora: neurological case histories from five neurological textbooks (referred to as *Textbook Corpus* (58–62), clinical synopses of neurological disease from the Online Mendelian Inheritance of Man (referred to as *OMIM Corpus*) (18), and neurology physician notes from the electronic health record of the University of Illinois at Chicago (referred to as *EHR Corpus*). The use of de-identified physician notes was approved by the Institutional Review Board of the University of Illinois at Chicago. Corpora were converted to plain text files and pre-processed using python. Email addresses, URLs, HTML, special characters, and unnecessary punctuation were removed using regular expressions in python. Contractions were replaced with the expanded form. Misspelled words, separated words, and hyphenated words were corrected manually using the spelling correction tool in Microsoft Word. Abbreviations were not edited. The pre-processed files were manually inspected for errors and converted to JSONL files.

### B. Text annotation

Signs and symptoms in JSONL files were annotated by a neurologist using the Prodigy annotation tool (63,64). An inter-rater reliability study with two other raters based on fifteen neurology notes showed an unadjusted agreement rate for text span annotation of 89% and a kappa statistic of 0.85 (65).

Each sign or symptom was tagged as a unigram, bigram, trigram, tetragram, extended, compound, or tabular concept. Unigrams were signs and symptoms of length one-word such as *alexia*, *hyperreflexia*, or *bradykinesia*. Bigrams were signs and symptoms of length two-words such as *double vision*, *facial weakness*, and *poor balance*. Trigrams were signs of symptoms of length three-words such as *absent ankle reflex*, *impaired hand dexterity*, or *weak ankle dorsiflexors*. Tetragrams were four-word signs and symptoms such as *relative afferent pupil defect* and *Hoffman sign was present*. Text spans were tagged as *extended* when signs and symptoms were more than four words, such as *hand grip was very weak* and *barely able to lift his legs off the bed*. Text spans were tagged as *compound* when more than one sign or symptom was combined in a single text span such as *decreased vibratory sensation, joint position, and pinprick below the knees*. *Tabular* concepts with separate columns for the right and left sides of the body were found only in the EHR notes. Examples of concepts in table form included biceps weakness represented as *[biceps strength 3 3]* (meaning that biceps strength was 3/5 on both right and left sides) or knee hyperreflexia represented as *[knee reflexes 4+ 4+]* (meaning that the knee reflex was 4+ on both right and left sides). Text span annotations were stored in an SQLite database and exported in JSONL format for further processing in the *spaCy* (Explosion, Berlin, Germany) python programming environment.

### C. NN model training and evaluation

Two neural network models were trained to recognize text spans that encoded clinical concepts in text corpora. Both models were based on NER pipelines. NER pipelines identify a named entity in a text span and assign the named entity to a predefined category. Each NN model assigned text spans to one of the seven defined categories of clinical concepts (unigram, bigram, trigram, tetragram, extended, compound, and tabular). For each corpus, 80% of the instances were used for training and 20% for evaluation. The baseline NN was the default spaCy named entity recognition model based on a four-layer convolutional neural network (CNN) that looks at four words on either side of each token using the NER pipeline and *tok2vec* with an initial learning rate $1\times 10^{-3}$. The standard word vectors included with spaCy were used for word embedding.

The second named entity recognition model was based on BERT (51). The BERT base model was implemented in spaCY (66) and consisted of 12 layers of transformer encoder, 12 attention heads, 786 hidden size, and 100 M parameters. The BERT model was pre-trained with publicly available weights and fine-tuned using our training set. We used the Adam optimizer with a learning rate of $5\times 10^{-5}$, $\beta_{1}=0.9$, $\beta_{2}=0.99$, a learning rate warm-up over the first 500 steps, and a linear decay learning rate. The dynamic batch size was set according to the longest sequence in the batch. The training was conducted over 20,000 steps. The mini-batch size dynamically changed according to the longest sequence in the batch. The largest padded size for batch sequences was 2,000, and the buffer was 256. A GELU activation function was used. For each corpus and each model, the F score, precision, and recall were computed (Table I).

**Table I.** Performance of CNN and BERT neural networks on concept extraction task.

Corpus	NN	F	Precision	Recall
EHR	CNN	57.5	65.6	51.2
	BERT	61.7	64.0	59.5
Textbook	CNN	69.0	70.1	67.9
	BERT	73.0	73.6	72.3
OMIM	CNN	76.2	78.8	73.7
	BERT	80.4	79.0	82.0

### D. Mapping text spans to concepts in the neuro-ontology (normalization)

Candidate text spans identified by the CNN and BERT models were mapped to neurological concepts in the target neuro-ontology. The neuro-ontology (57) is a hierarchical ontology with 1,600 concepts constructed with the Protégé ontology editor (67). All concepts map to terms and CUIs (unique concept identifiers) from the UMLS (20). The highest levels of neuro-ontology correspond to the main elements of the neurological examination: mental status, cranial nerves, motor, sensory, reflexes, and symptoms. The neuro-ontology is available for download in CSV or OWL format at the National Center for Biomedical Ontologies BioPortal (https://bioportal.bioontology.org/ontologies/NEO).

We manually created a look-up table by mapping 3,500 potential target phrases to concepts in the neuro-ontology. Similarities between the candidate text spans (from either the CNN or BERT models) and target phrases in the lookup table were calculated using the *doc.similarity* method from spaCy (66). Both the candidate text span and the target phrase were converted to *doc* objects using the spaCy NLP pipeline (https://spacy.io/api/doc/#similarity), which converts each token in the phrase into a word vector. The similarity is the cosine distance between the word vectors from the two phrases and ranges between 0.0 (least similar) and 1.0 (most similar). We mapped the candidate text span to its most similar target text span in the look-up table and retrieved the corresponding concept name and UMLS CUI from the neuro-ontology (57).

## III. Results

### A. Writing style and accuracy varied by corpus

**Figure 2:**
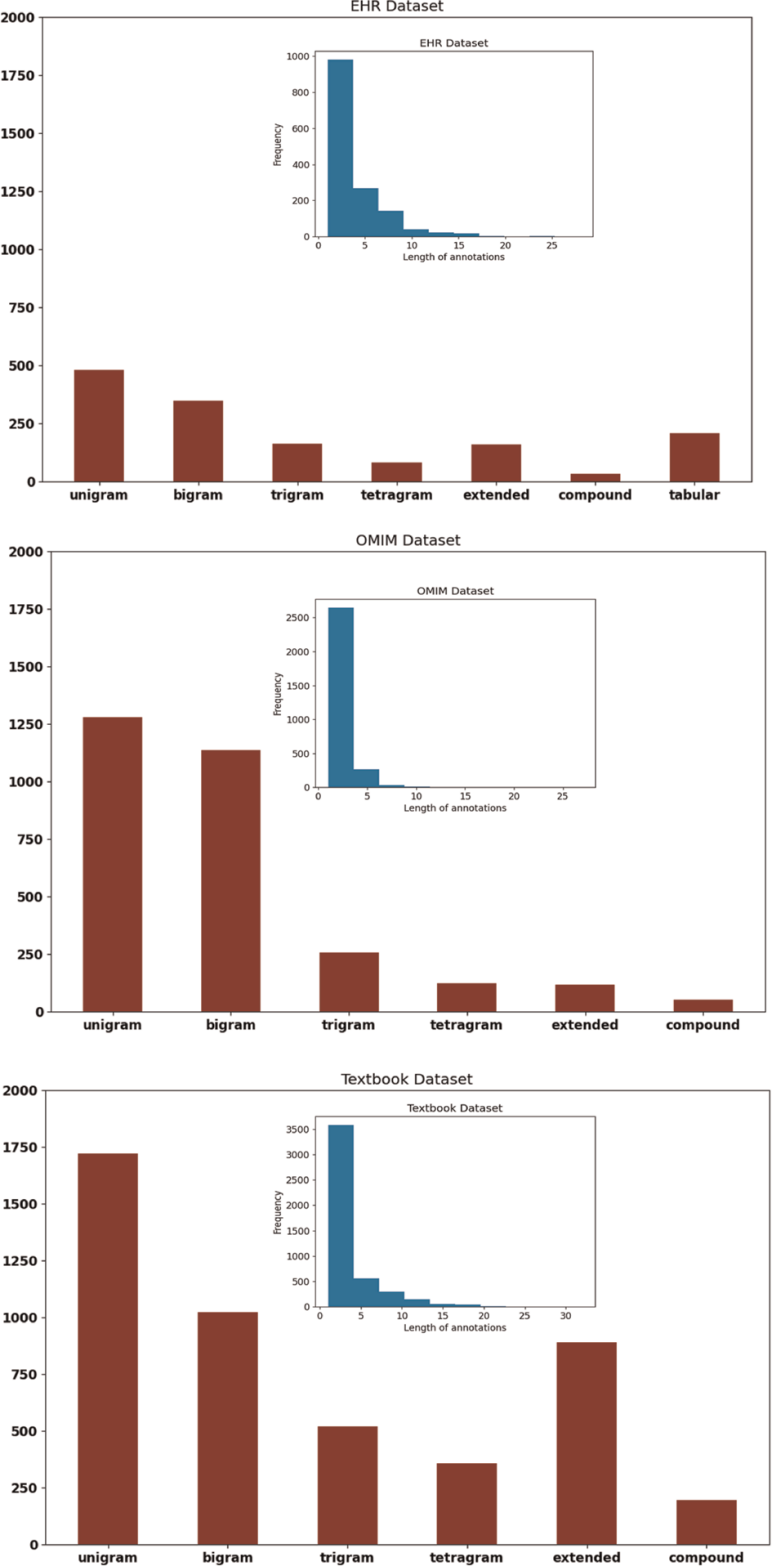
Text spans that identified clinical concepts were longer in the EHR corpus and shortest in the OMIM corpus (see blue inset histograms). Proportionately, the OMIM corpus used the most unigrams and bigrams as compared to the EHR corpus and the Textbook corpus (see red bar charts).

The OMIM corpus used more unigrams and digrams to encode signs and symptoms and had shorter spans of text annotations than the EHR corpus or the Textbook corpus (Figure 2). The length of annotations (histogram insets, Figure 2) was longer for the EHR corpus. Extended annotations were more frequent in the EHR corpus and Textbook corpus. Only the EHR corpus had tabular annotation (clinical concepts expressed in table format). Performance on the concept identification task differed by corpus; F, precision, and recall were highest for the OMIM corpus and lowest for the EHR corpus (Table 1).

### B. Performance of NER model decreased with the increasing text span length

**Figure 3:**
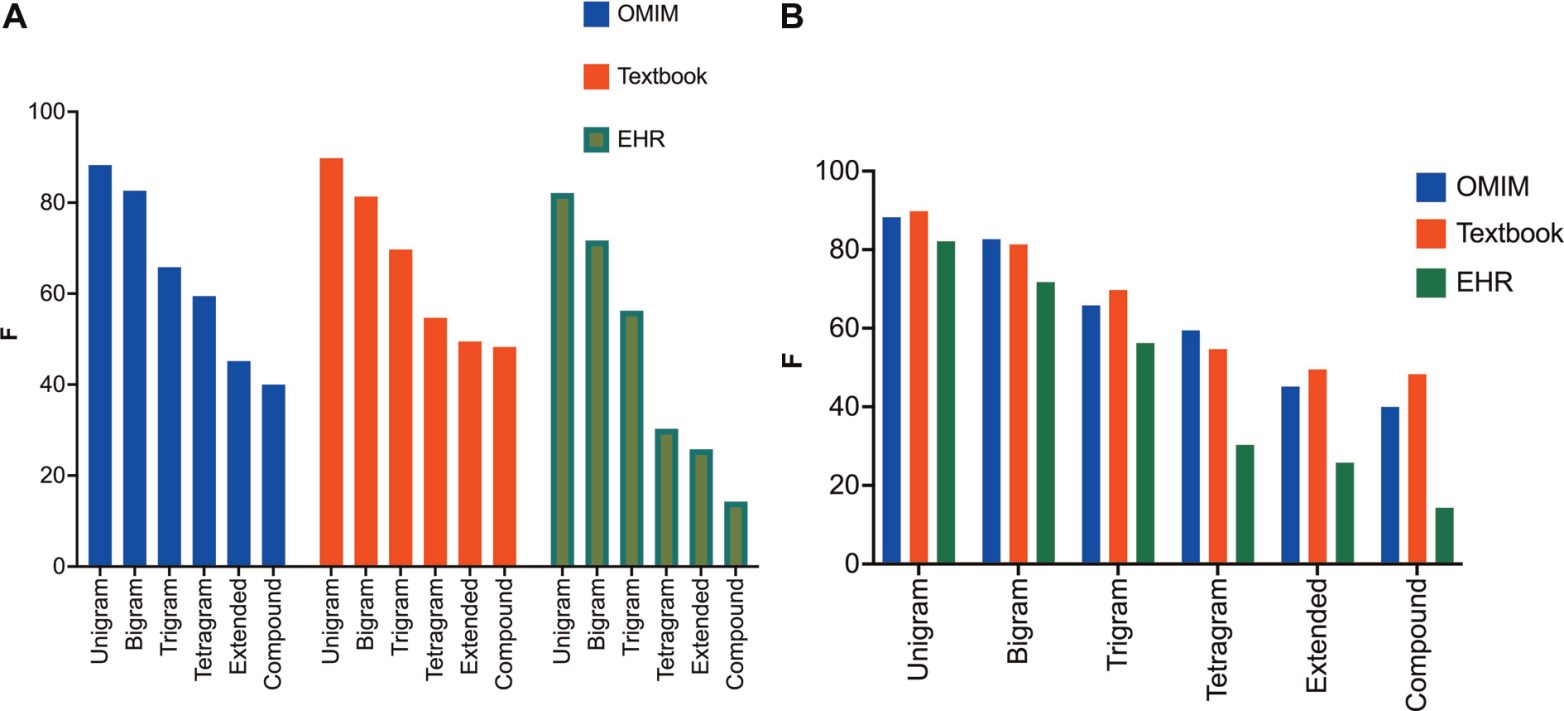
(**A**) F values for the BERT NER model sorted by text span label type. The BERT NER model performs similarly on the three corpora for unigrams and bigrams, but F values lag for the EHR corpora for the tetragrams, extended text spans, and compound text spans. Note that the BERT NER model performs significantly worse on the EHR corpus for tetragrams, extended text spans, and compound text spans when compared to the Textbook or OMIM corpus. (**B**) F values for the BERT NER model sorted by corpus. All three corpora show the same pattern with declining F values with the increasing length of the text span.

For all three corpora, the recognition of clinical concepts as measured by F scores was better for shorter text spans (Figures 3A,B). This applied to both the CNN and the BERT models for concept identification (Table 1). F was highest for unigrams (one-word concepts like *ataxia*, *diplopia*, *aphasia*) for all three corpora. In general, performance on bigrams was better than trigrams, and performance on trigrams was better than tetragrams. Performance tended to be worse for text spans greater than four words (extended), or text spans with compound constructions such as *weakness of the biceps, triceps, and deltoids*.

### C. Performance varied by neural network model

For all three corpora, BERT outperformed the CNN neural network for the recall of clinical concepts. Precision in clinical concept identification was about the same for all three corpora when BERT was compared to the CNN model (Table 1).

## IV. Discussion

Named entity recognition models based on deep learning can recognize neurologic signs and symptoms in the biomedical literature and electronic health records (Table 1). Previous work has shown that BERT outperforms CNNs on recognizing drugs and diseases in annotated test corpora (52,55). We extend these observations to demonstrate the superiority of BERT over CNNs for recognizing neurological signs and symptoms in electronic health records and biomedical literature.

A significant finding was that the accuracy of recognition of signs and symptoms fell with increasing text span length (Figures 3A,B). Increased variability in longer text spans likely poses greater difficulty for NER pipelines, regardless of whether they are based on linguistic/symbolic methods like MetaMap or deep learning like BERT or CNNs. Longer text spans are more likely to be descriptions of named entities (e.g., “the patient fell to the left when standing with eyes closed” rather than more concise named entities themselves (e.g., “Romberg sign positive”). Normalization of longer text spans (mapping to suitable concepts in the ontology) may pose additional challenges. The successful mapping (normalization) of “wavering with eyes closed” to “Romberg sign positive” may require vectorization (word embedding) of terms in an ontology, as well as the synonyms and definitions of these terms (48,55).

Another significant observation was that recall of neurologic signs and symptoms was lower in the EHR corpus than in the OMIM corpus or Textbook corpus. The Textbook and the OMIM corpus were written by professional writers and had undergone careful editing and correction. The EHR corpus was written by physicians who were not professional writers. The EHR corpus was marred by irregular spelling, irregular abbreviations, typographical errors, grammatical errors, and other irregularities absent from the OMIM corpus and the Textbook corpus. Others have noted the high frequency of irregular abbreviations, spelling, grammatical, and other writing errors in the clinical notes created by physicians (68–72) The general approach of the writers of the OMIM corpus was brevity. OMIM writers tended to use lists of clinical concepts such as “the patient had optic disk pallor, miosis, anisocoria, and a relative afferent pupil defect.” The general approach of the writers of the Textbook corpus was didactic and explanatory so that a relative afferent pupil defect might be described as “the swinging flashlight test was abnormal and the pupil dilated when the light was placed over the abnormal pupil and the pupil constricted when the light was moved to the normal pupil.” The EHR corpus was characterized by brevity but irregular spellings, abbreviations, and syntax so that the same patient might be described as “RAPD present on R.”

The lower accuracy for recognition of signs and symptoms in the EHR corpus (physician notes) deserves further comment. One way to improve automated recognition of signs and symptoms in physician notes is to encourage them to use structured rather than unstructured documentation (73). However, given physician burnout associated with clinical documentation (74), and physician distaste for structured documentation (75), it seems unlikely that physicians will adopt structured documentation for recording signs and symptoms. Furthermore, given that by training, physicians are often asked to describe findings rather than name findings, it seems unlikely that physicians can be converted to using short names instead of lengthy descriptions of signs and symptoms. Rather, improvements in NLP are needed to identify better clinical concepts held as lengthier texts spans or represented as descriptions of named entities rather than as the named entity itself.

NLP models that extract clinical concepts from free text must recognize negation successfully. The sentence “the patient has ataxia” has a clinical concept whereas the sentence “ataxia is absent” denies ataxia (76–78). Negation makes it difficult to determine if a sign or symptom is present and suggests that strategies based on regular expressions (REGEX) will fail. The patient who complains of tremor, who is tremulous, or is observed to have a tremor must be distinguished from the patient who denies tremor, is not tremulous, or has no tremor. MetaMap uses the NEGEX algorithm to recognize negation (27). We relied on examples to train the neural networks to recognize negated concepts for our BERT and CNN models. Further work is needed on handling negated concepts accurately and efficiently (77). Another challenge is word disambiguation (79). The sentence the “patient has had a fall” may contain a valid neurological concept, whereas the sentence “the patient was seen in the Fall” does not. Word disambiguation is another area of continuing research in NLP (79).

This study has several limitations. The study was limited to the domain of neurology (neurological signs and symptoms). Furthermore, the text span annotations were done by a single annotator. We have planned an inter-rater agreement study (65). We limited the target ontology to 1,600 neurological concepts. Whether our methods can be generalized to more complex domains and larger ontologies is uncertain. Although we achieved a recall of 80% to 90% with shorter text span lengths, the recall was lower for longer text span lengths. To make automated high throughput neuro-phenotyping practical, we estimate that a recall of at least 90% is needed depending on the application (i.e., research versus patient care). Identifying clinical concepts in complex grammatical structures remains challenging for even the best NLP algorithms. For example, identifying the concepts *biceps weakness*, *triceps weakness*, and *hand weakness* in the sentence *the patient had 3+/5 strength in the biceps, 2+/5 strength in the triceps, and 1/5 hand grip strength* remains problematic. Efficient NLP algorithms that simplify grammar and syntax are an area of evolving research (80,81). Another limitation of the study is the small corpus used for training. Our NER models would likely have improved with more training annotations.

In conclusion, given the burden of physician documentation (74), patient signs and symptoms will likely continue in electronic health records as unstructured text. The automated identification of these signs and symptoms is critical to the success of deep phenotyping, and precision medicine initiatives (5,6). Advances in NLP based on word embedding and deep learning make the automated identification of signs and symptoms in unstructured text increasingly feasible.

## Data availability statement

The original contributions presented in the study are included in the article/supplementary material, further inquiries can be directed to the corresponding author/s.

## Ethics statement

The studies involving human participants were reviewed and approved by Institutional Review Board of the University of Illinois at Chicago. The patients/participants provided their written informed consent to participate in this study.

## Author’s contributions

Concept and design by SA and DBH. Model parameters and computations by SA. Data interpretation, drafting, revising, and final approval by SA, DBH, and DCW II.All authors contributed to the article and approved the submitted version.

## Funding

The research was partially sponsored by the Mary K. Finley Missouri Endowment, the Missouri S&T Intelligent Systems Center, the National Science Foundation, and the Leonard Wood Institute in cooperation with the U.S. Army Research Laboratory. It was accomplished under Cooperative Agreement Number W911NF-14-2-0034. The views, opinions, findings, recommendations, or conclusions contained in this document are those of the authors. They should not be interpreted as representing the views or official policies expressed or implied by the Leonard Wood Institute, the Army Research Laboratory, the National Science Foundation, or the U.S. Government. The U.S. Government is authorized to reproduce and distribute reprints for Government purposes notwithstanding any copyright notation hereon.

## References

[1] Krauthammer M Nenadic G. Term identification in the biomedical literature. J Biomed Inform. (2004) 37:512–26. 10.1016/j.jbi.2004.08.004 15542023

[2] Office of the National Coordinator for Health Information Technology. Adoption of electronic health records by hospital service type 2019–2021, Health IT Quick Stat #60 (2022). Available from: https://www.healthit.gov/data/quickstats/adoption-electronic-health-records-hospital-service-type-2019-2021.

[3] Banda JM Seneviratne M Hernandez-Boussard T Shah NH. Advances in electronic phenotyping: from rule-based definitions to machine learning models. Annu Rev Biomed Data Sci. (2018) 1:53. 10.1146/annurev-biodatasci-080917-013315 31218278 PMC6583807

[4] Tenopir C Grayson M Zhang Y Ebuen M King DW Boyce PB. Patterns of journal use by scientists through three evolutionary phases. D-Lib (2003) 9:1–15. 10.1045/may2003-king

[5] Collins FS Varmus H. A new initiative on precision medicine. N Engl J Med. (2015) 372:793–5. 10.1056/NEJMp1500523 25635347 PMC5101938

[6] Robinson PN. Deep phenotyping for precision medicine. Hum Mutat. (2012) 33:777–80. 10.1002/humu.22080 22504886

[7] Fu S Chen D He H Liu S Moon S Peterson KJ, et al. Clinical concept extraction: a methodology review. J Biomed Inform. (2020) 109:103526. 10.1016/j.jbi.2020.103526 PMC7746475 32768446

[8] Esteva A Robicquet A Ramsundar B Kuleshov V DePristo M Chou K, et al. A guide to deep learning in healthcare. Nat Med. (2019) 25:24–9. 10.1038/s41591-018-0316-z 30617335

[9] Chowdhary K. Natural language processing. Fundam Artif Intell. (2020):603–49.

[10] Hirschberg J Manning CD. Advances in natural language processing. Science. (2015) 349:261–6. 10.1126/science.aaa8685 26185244

[11] Islam MA, Anik MSH, Islam ABMAA. Towards achieving a delicate blending between rule-based translator, neural machine translator. Neural Comput Appl. (2021) 33:12141–67. 10.1007/s00521-021-05895-x

[12] Islam MA Mukta MSH Olivier P Rahman MM. Comprehensive guidelines for emotion annotation. *Proceedings of the 22nd ACM International Conference on Intelligent Virtual Agents*, 2022 Sep. New York, NY, USA: Association for Computing Machinery (2022). p. 1–8.

[13] Mohammad S. A practical guide to sentiment annotation: challenges, solutions. *Proceedings of the 7th Workshop on Computational Approaches to Subjectivity, Sentiment, Social MEDIA Analysis*, 2016 Jun. San Diego, California: Association for Computational Linguistics (2016). p. 174–9.

[14] Hasan HM Islam MA Hasan MT Hasan MA Rumman SI Shakib MN. A spell-checker integrated machine learning based solution for speech to text conversion. *2020 Third International Conference on Smart Systems and Inventive Technology (ICSSIT)* (2020). p. 1124–30.

[15] Gonzalez-Hernandez G Sarker A O’Connor K Savova G. Capturing the patient’s perspective: a review of advances in natural language processing of health-related text. Yearb Med Inform. (2017) 26:214–27. 10.15265/IY-2017-029 29063568 PMC6250990

[16] Bird S Klein E Loper E. Natural language processing with python. Sebastopol, CA: O’Reilly Media (2009). Available from: https://www.nltk.org/book/.

[17] SNOMED CT. *NCBO BioPortal* (2022). Available from: https://bioportal.bioontology.org/ontologies/SNOMEDCT/ (Accessed October 5, 2022).

[18] Online Mendelian Inheritance in Man. *NCBO BioPortal* (2022). Available from: https://bioportal.bioontology.org/ontologies/OMIM (Accessed October 5, 2022).

[19] Human Phenotype Ontology. *NCBO BioPortal* (2022). Available from: https://bioportal.bioontology.org/ontologies/HP (Accessed October 5, 2022).

[20] UMLS Metathesaurus Browser. *National Library of Medicine* (2022). Available from: https://uts.nlm.nih.gov/uts/umls/home (Accessed October 5, 2021).

[21] Eltyeb S Salim N. Chemical named entities recognition: a review on approaches, applications. J Cheminform. (2014) 6:1–12. 10.1186/1758-2946-6-17 24834132 PMC4022577

[22] Quimbaya AP Múnera AS Rivera RAG Rodríguez JCD Velandia OMM Peña AAG, et al. Named entity recognition over electronic health records through a combined dictionary-based approach. Procedia Comput Sci. (2016) 100:55–61. 10.1016/j.procs.2016.09.123

[23] Hirschman L Morgan AA Yeh AS. Rutabaga by any other name: extracting biological names. J Biomed Inform. (2002) 35:247–59. 10.1016/S1532-0464(03)00014-5 12755519

[24] Uzuner Ö South BR Shen S DuVall SL. 2010 i2b2/VA challenge on concepts, assertions,, relations in clinical text. J Am Med Inform Assoc. (2011) 18:552–6. 10.1136/amiajnl-2011-000203 21685143 PMC3168320

[25] Funk C Baumgartner W Garcia B Roeder C Bada M Cohen KB, et al. Large-scale biomedical concept recognition: an evaluation of current automatic annotators and their parameters. BMC Bioinf. (2014) 15:1–29. 10.1186/1471-2105-15-59 PMC4015610 24571547

[26] Shah NH Bhatia N Jonquet C Rubin D Chiang AP Musen MA. Comparison of concept recognizers for building the open biomedical annotator. BMC Bioinf. (2009) 10:1–9. 10.1186/1471-2105-10-S2-S1 PMC2745685 19761568

[27] Aronson AR Lang FM. An overview of MetaMap: historical perspective and recent advances. J Am Med Inform Assoc. (2010) 17:229–36. 10.1136/jamia.2009.002733 20442139 PMC2995713

[28] Lindberg DA Humphreys BL McCray AT. The unified medical language system. Yearb Med Inform. (1993) 2:41–51. 10.1055/s-0038-1637976 PMC6693523 27668467

[29] Bodenreider O. The unified medical language system (UMLS): integrating biomedical terminology. Nucleic Acids Res. (2004) 32:D267–70. 10.1093/nar/gkh061 14681409 PMC308795

[30] Hier DB Yelugam R Azizi S Carrithers MD Wunsch II DC. High throughput neurological phenotyping with MetaMap. Eur Sci J. (2022) 18:37–49. 10.19044/esj.2022.v18n4p37

[31] Wang Y Wang L Rastegar-Mojarad M Moon S Shen F Afzal N, et al. Clinical information extraction applications: a literature review. J Biomed Inform. (2018) 77:34–49. 10.1016/j.jbi.2017.11.011 29162496 PMC5771858

[32] Sevenster M Van Ommering R Qian Y. Automatically correlating clinical findings and body locations in radiology reports using MedLEE. J Digit Imaging. (2012) 25:240–9. 10.1007/s10278-011-9411-0 21796490 PMC3295967

[33] Savova GK Masanz JJ Ogren PV Zheng J Sohn S Kipper-Schuler KC, et al. Mayo clinical text analysis and knowledge extraction system (cTAKES): architecture, component evaluation and applications. J Am Med Inform Assoc. (2010) 17:507–13. 10.1136/jamia.2009.001560 20819853 PMC2995668

[34] Friedman C Shagina L Lussier Y Hripcsak G. Automated encoding of clinical documents based on natural language processing. J Am Med Inform Assoc. (2004) 11:392–402. 10.1197/jamia.M1552 15187068 PMC516246

[35] Friedman C Shagina L Socratous SA Zeng X. A web-based version of MedLEE: a medical language extraction and encoding system. *Proceedings of the AMIA Annual Fall Symposium*. American Medical Informatics Association (1996). p. 938.

[36] Friedman C. A broad-coverage natural language processing system. *Proceedings of the AMIA Symposium*. American Medical Informatics Association. (2000). p. 270–4. PMC2243979 11079887

[37] Huang Z Xu W Yu K. Bidirectional LSTM-CRF models for sequence tagging [Preprint] (2015). Available at: http://arxiv.org/1508.01991.

[38] Lample G Ballesteros M Subramanian S Kawakami K Dyer C. Neural architectures for named entity recognition [Preprint] (2016). Available at: http://arxiv.org/1603.01360.

[39] Chiu JP Nichols E. Named entity recognition with bidirectional LSTM-CNNs. Trans Assoc Comput Linguist. (2016) 4:357–70. 10.1162/tacl-a-00104

[40] Peters ME Ammar W Bhagavatula C Power R. Semi-supervised sequence tagging with bidirectional language models [Preprint] (2017). Available at: http://arxiv.org/1705.00108.

[41] Vani A Jernite Y Sontag D. Grounded recurrent neural networks [Preprint] (2017). Available at: http://arxiv.org/1705.08557.

[42] Liu Z Tang B Wang X Chen Q. De-identification of clinical notes via recurrent neural network and conditional random field. J Biomed Inform. (2017) 75:S34–S42. 10.1016/j.jbi.2017.05.023 PMC5705329 28579533

[43] Habibi M Weber L Neves M Wiegandt DL Leser U. Deep learning with word embeddings improves biomedical named entity recognition. Bioinformatics. (2017) 33:i37–i48. 10.1093/bioinformatics/btx228 28881963 PMC5870729

[44] Dehghan A Kovacevic A Karystianis G Keane JA Nenadic G. Combining knowledge-and data-driven methods for de-identification of clinical narratives. J Biomed Inform. (2015) 58:S53–9. 10.1016/j.jbi.2015.06.029 26210359 PMC4976126

[45] Hochreiter S Schmidhuber J. Long short-term memory. Neural Comput. (1997) 9:1735–80. 10.1162/neco.1997.9.8.1735 9377276

[46] Lafferty J McCallum A Pereira FC. Conditional random fields: probabilistic models for segmenting and labeling sequence data. *Proceedings of the 18th International Conference on Machine Learning 2001*. San Francisco, CA, USA: Morgan Kaufmann Publishers Inc. (2001).

[47] Gehrmann S Dernoncourt F Li Y Carlson ET Wu JT Welt J, et al. Comparing deep learning and concept extraction based methods for patient phenotyping from clinical narratives. PLoS ONE. (2018) 13:e0192360. 10.1371/journal.pone.0192360 29447188 PMC5813927

[48] Arbabi A Adams DR Fidler S Brudno M, et al. Identifying clinical terms in medical text using ontology-guided machine learning. JMIR Med Inform. (2019) 7:e12596. 10.2196/12596 31094361 PMC6533869

[49] Groza T Köhler S Doelken S Collier N Oellrich A Smedley D, et al. Automatic concept recognition using the human phenotype ontology reference and test suite corpora. Database. (2015) 2015:1–13. 10.1093/database/bav005 PMC4343077 25725061

[50] Vaswani A Shazeer N Parmar N Uszkoreit J Jones L Gomez AN, et al. Attention is all you need. Adv Neural Inf Process Syst. (2017) 30:5998–6008.

[51] Devlin J Chang MW Lee K Toutanova K. BERT: pre-training of deep bidirectional transformers for language understanding [Preprint] (2018). Available at: http://arxiv.org/1810.04805.

[52] Zhu R Tu X Huang JX. Utilizing BERT for biomedical, clinical text mining. *Data Analytics in Biomedical Engineering, Healthcare*. Elsevier (2021). p. 73–103. Available from: 10.1016/B978-0-12-819314-3.00005-7

[53] Yu X Hu W Lu S Sun X Yuan Z. Biobert based named entity recognition in electronic medical record. *2019 10th international conference on information technology in medicine and education (ITME)*. New York NY: IEEE (2019). p. 49–52.

[54] Lee J Yoon W Kim S Kim D Kim S So CH, et al. Biobert: a pre-trained biomedical language representation model for biomedical text mining. Bioinformatics. (2020) 36:1234–40. 31501885 10.1093/bioinformatics/btz682 PMC7703786

[55] Ji Z Wei Q Xu H. Bert-based ranking for biomedical entity normalization. AMIA Summits Transl Sci Proc. (2020) 2020:269. 32477646 PMC7233044

[56] Weng C Shah NH Hripcsak G. Deep phenotyping: embracing complexity and temporality-towards scalability, portability, and interoperability. J Biomed Inform. (2020) 105:103433. 10.1016/j.jbi.2020.103433 32335224 PMC7179504

[57] Hier DB Brint SU. A neuro-ontology for the neurological examination. BMC Med Inform Decis Mak. (2020) 20:1–9. 10.1186/s12911-020-1066-7 32131804 PMC7057564

[58] Gondolo T. Neurology study guide: oral board examination review. Cham Switzerland: Springer Nature (2005).

[59] Ubogu EE. Neurology oral boards review. New York NY: Humana Press (2005).

[60] Alpert JN. The neurologic diagnosis: a practical bedside approach. Cham Switzerland: Springer (2018).

[61] Kung D Nguyen T. Absolute case-based neurology review. Oxford UK: Springer (2019).

[62] Macleod M Pal S Simpson M. Neurology clinical cases uncovered. San Francisco CA: Wiley-Blackwell (2011).

[63] Neves M Ševa J. An extensive review of tools for manual annotation of documents. Brief Bioinformatics. (2021) 22:146–63. 10.1093/bib/bbz130 31838514 PMC7820865

[64] Montani I Honnibal M. Prodigy: a new annotation tool for radically efficient machine teaching. Artif Intell. (2018). Available from: https://explosion.ai/blog/prodigy-annotation-tool-active-learning.29731511

[65] Oommen C Howlett-Prieto Q Carrithers MD Hier DB. Inter-Rater Agreement for the Annotation of Neurologic Concepts in Electronic Health Records. *medRxiv* (2022). Available from: 10.1101/2022.11.16.22282384. PMC10294690 37383943

[66] Vasiliev Y. Natural language processing with Python and Spacy. San Francisco CA: No Starch Press (2020).

[67] Noy NF McGuinness DL. Ontology development 101: a guide to creating your first ontology. *Stanford Knowledge Systems Laboratory Technical Report KSL-01-05* (2001).

[68] Assale M Dui LG Cina A Seveso A Cabitza F. The revival of the notes field: leveraging the unstructured content in electronic health records. Front Med. (2019) 6:66. 10.3389/fmed.2019.00066 PMC6478793 31058150

[69] Shilo G Shilo L. Writing style of young physicians in the computer and internet era. Int J Med Educ. (2014) 5:82. 10.5116/ijme.534a.a3e2 25341216 PMC4207182

[70] Pagano MP Mair D. Writing medical records. J Tech Writ Commun. (1986) 16:331–41. 10.2190/WY9T-634E-V2JT-JDVQ

[71] Zisowitz ML. Teaching medical students and physicians to write. Acad Med. (1964) 39:481–4. 14143996

[72] Hamiel U Hecht I Nemet A Pe’er L Man V Hilely A, et al. Frequency, comprehension and attitudes of physicians towards abbreviations in the medical record. Postgrad Med J. (2018) 94:254–8. 10.1136/postgradmedj-2017-135515 29540451

[73] Rosenbloom ST Denny JC Xu H Lorenzi N Stead WW Johnson KB. Data from clinical notes: a perspective on the tension between structure and flexible documentation. J Am Med Inform Assoc. (2011) 18:181–6. 10.1136/jamia.2010.007237 21233086 PMC3116264

[74] Thomas Craig KJ Willis VC Gruen D Rhee K Jackson GP. The burden of the digital environment: a systematic review on organization-directed workplace interventions to mitigate physician burnout. J Am Med Inform Assoc. (2021) 28:985–97. 10.1093/jamia/ocaa301 33463680 PMC8068437

[75] Han H Lopp L. Writing and reading in the electronic health record: an entirely new world. Med Educ Online. (2013) 18:18634. 10.3402/meo.v18i0.18634 PMC3566375 23394976

[76] Shivade C de Marneffe MC Fosler-Lussier E Lai AM. Extending negex with kernel methods for negation detection in clinical text. *Proceedings of the Second Workshop on Extra-Propositional Aspects of Meaning in Computational Semantics (ExProM 2015)* (2015). p. 41–46.

[77] Wu S Miller T Masanz J Coarr M Halgrim S Carrell D, et al. Negation’s not solved: generalizability versus optimizability in clinical natural language processing. PLoS ONE. (2014) 9:e112774. 10.1371/journal.pone.0112774 25393544 PMC4231086

[78] Elkin PL Brown SH Bauer BA Husser CS Carruth W Bergstrom LR, et al. A controlled trial of automated classification of negation from clinical notes. BMC Med Inform Decis Mak. (2005) 5:1–7. 10.1186/1472-6947-5-13 15876352 PMC1142321

[79] Navigli R. Word sense disambiguation: a survey. ACM Comput Surv. (2009) 41:1–69. 10.1145/1459352.1459355

[80] Shardlow M. A survey of automated text simplification. Int J Adv Comput Sci Appl. (2014) 4:58–70.

[81] Al-Thanyyan SS Azmi AM. Automated text simplification: a survey. ACM Comput Surv. (2021) 54:1–36. 10.1145/3442695

